# Skin-Interfaced Flexible Sensors for Continuous Monitoring of Thermal, Mechanical, Electrical, and Respiratory Signals

**DOI:** 10.3390/bios16100551

**Published:** 2026-10-01

**Authors:** You Zhou, Dongfen Li, Wentao Ni, Bowen Deng, Weiqian Liang, Jianyuan Tang, Yue Huang

**Affiliations:** College of Computer and Network Security, Chengdu University of Technology, 1#, Dongsanlu, Erxianqiao, Chengdu 610059, China

**Keywords:** skin-interfaced flexible sensors, continuous physiological monitoring, measurement validity, skin–device interface, wearable electronics

## Abstract

Skin-interfaced flexible sensors enable physiological monitoring during daily activities, sleep, and rehabilitation, but a sensitive device or a clear waveform does not by itself establish the validity of the physiological information obtained. Many existing reviews emphasize sensor materials, fabrication, device design, and sensing performance. This review instead examines how physiological information is preserved or altered from its origin to its eventual interpretation. We focus on flexible physical sensors for skin temperature, mechanical activity, surface biopotentials, electrodermal activity, and respiration-related monitoring. The scope is restricted to selected physical and skin surface electrical sensing pathways, while optical, biochemical, and implantable technologies are not reviewed in detail. The analysis links the physical quantity available at the skin to its transmission across the skin–device interface, sensor response, signal fidelity, and the physiological output that the measurement can reasonably support. This approach distinguishes direct measurements from derived or clinically interpreted quantities and shows where additional reference-based validation becomes necessary. Across the reviewed modalities, major limitations arise when interface conditions or environmental and mechanical interference alter the relationship between the physiological source and the recorded signal. Reliable monitoring therefore depends on preserving this relationship under intended wearing conditions and demonstrating agreement with references appropriate to the claimed output. By focusing on this measurement process, the review complements materials- and device-oriented surveys and provides a basis for judging flexible sensors according to the physiological information that their measurements can support.

## 1. Introduction

With the aging population and the increasing burden of chronic non-communicable diseases, health management has gradually shifted from intermittent diagnosis and treatment after symptoms to long-term state assessment and early risk identification. Noncommunicable diseases caused at least 43 million deaths in 2021, accounting for approximately 75% of all non-pandemic-related deaths worldwide [1]. Their chronic and fluctuating courses make it difficult for assessments conducted at isolated clinical time points to fully represent an individual’s health status. The standard equipment in the hospital can provide relatively accurate measurement results, but its use is usually limited by the site, time and operating conditions, so it is difficult to continuously capture the short-term physiological changes during sleep, daily activities or rehabilitation. Wearable sensing technology extends data collection to the daily environment, which provides new measurement conditions for establishing an individual baseline and observing the dynamic evolution of physiological indicators.

Skin is an important non-invasive interface for continuously obtaining human physiological information. Heat transfer, tissue movement and electrical activity inside the body can form measurable physical changes on the body surface, and some complex physiological processes can also be indirectly characterized by the skin response caused by them. By reducing the bending stiffness of the device and the mechanical mismatch between the device and the skin, the flexible sensor can maintain contact for a long time with less wearing burden, thus improving the signal acquisition under dynamic conditions. The “Epidermal Electronics” proposed by Kim et al. enables the electronic system to complete the body surface signal acquisition under conditions close to the skin mechanical characteristics by reducing the device thickness, bending stiffness and area density at the same time, and then the related research is gradually extended from a single flexible sensor to a skin-mounted multi-parameter monitoring system [2]. Recent systems further extend this progression toward battery-free epidermal networks, cross-modal sensing, and integrated respiratory platforms, showing that current development increasingly depends on coordinated interface mechanics, signal separation, wireless integration, and physiological validation rather than sensor sensitivity alone [3,4,5,6].

However, improved conformity does not ensure that a recorded signal accurately represents the physiological quantity of interest. Before reaching the sensing element, physiological information may be altered by tissue transmission, anatomical location, skin condition, and changes in device attachment. The sensor itself usually records a local physical or electrical quantity rather than the physiological or clinical variable ultimately reported. The validity of a derived metric therefore depends on whether the relationship between the original physiological process and the recorded quantity remains sufficiently defined under the intended conditions of use.

This review focuses on selected flexible sensing pathways that acquire physical or electrical physiological information at or near the body surface. Section 2, Section 3 and Section 4 examine thermal, mechanical, and skin surface electrical measurements, whereas Section 5 addresses respiratory monitoring using several wearable-accessible proxy signals. Optical sensing is not reviewed in detail because its signal formation and interpretation require explicit treatment of light propagation, tissue optical properties, and photodetection. Recent optical and visually readable electronic skin concepts also illustrate the broader development of flexible tactile sensing, but these approaches are not used here as evidence for the physiological sensing pathways reviewed in detail [7]. Biochemical and implantable sensing systems are also outside the present scope because they involve different target measurands or measurement interfaces. This organization defines the scope of the present review and is not intended as an exhaustive classification of skin-interfaced sensing technologies.

A skin-interfaced sensor operates as part of a measurement system rather than as an isolated sensing material. Mechanical conformity, interconnection design, packaging, and local electronics can alter how the target signal reaches the sensing element or how the resulting output is recorded. These effects become especially important in untethered systems, where integration of readout, power, and communication components can modify the mechanical and thermal conditions at the skin. Materials and structures are therefore discussed in this review when they affect signal transmission, transduction, or measurement stability rather than being classified independently.

Existing reviews have established a broad understanding of flexible sensor materials, device design, fabrication, and wearable applications [8,9]. These perspectives are essential for explaining how sensing performance is produced at the device level, but they do not necessarily organize the literature around the relationship between a physiological source and the health-related quantity eventually reported. The present review addresses this relationship by distinguishing the quantity available at the skin from the device output and from the physiological metric derived from that output. This distinction is consistent with the V3 framework, which separates verification from analytical validation and clinical validation according to the type of evidence required for a digital measurement technology [10,11,12]. The reviewed studies are therefore assessed not only by sensor response but also by whether signal transmission, measurement fidelity, and reference-based evidence support the intended physiological interpretation.

**Figure 1:**
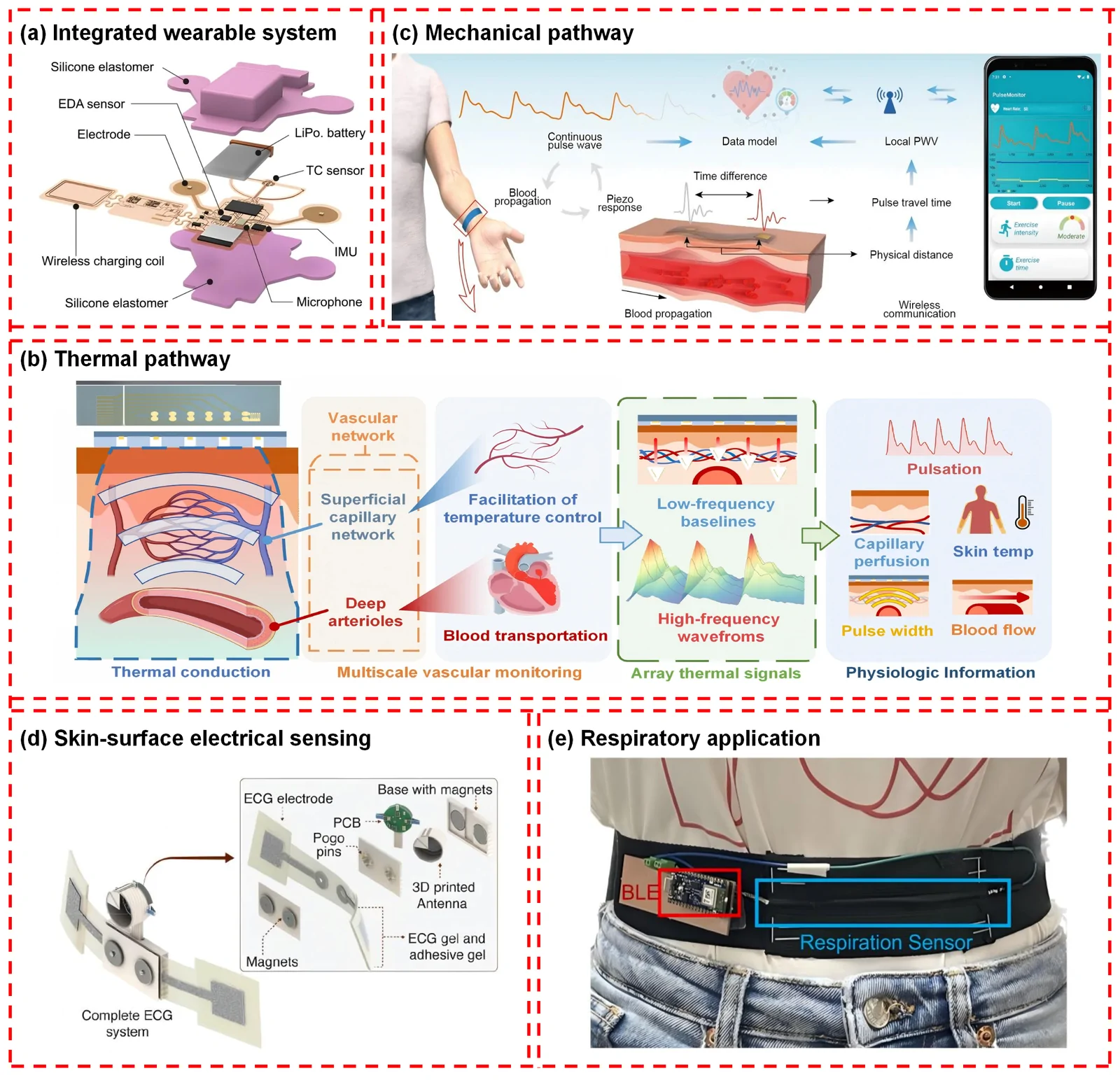
Scope and measurement organization of skin-interfaced flexible sensing for physiological monitoring. (**a**) Exploded architecture of an integrated skin-interfaced multimodal sensing system, illustrating the integration of sensing elements with flexible electronics and wearable system components. Adapted from [13]. (**b**) Thermal sensing pathway linking vascular activity to skin-mediated thermal responses and physiological information detected by a flexible thermal array. Adapted from [14]. (**c**) Wearable mechanical sensing pathway in which arterial motion is converted into piezoelectric signals and subsequently processed to obtain cardiovascular measurements. Adapted from [15]. (**d**) Integration of skin-contact electrocardiography (ECG) electrodes with compact wearable readout electronics for acquisition of skin surface electrical signals. Adapted from [16]. (**e**) Respiration band positioned on the abdomen for detection of breathing-related deformation, illustrating the use of a local mechanical proxy for respiratory monitoring. Adapted from [17].

The review uses a common measurement sequence to examine the included sensing modalities. A physiological process first produces a physical or electrical signal that becomes accessible at the skin, and this signal may be modified before reaching the sensing element by tissue properties and the skin–device interface. Sensor transduction then produces the recorded output, whose fidelity depends on whether the target signal can be separated from interference, drift, and loss of coupling. Physiological interpretation is considered only after these preceding relationships have been established, with the required validation determined by the output being claimed. Representative systems illustrate how this measurement sequence differs across sensing modalities. In an integrated multimodal platform, sensing elements, flexible electronics, and supporting components operate as a complete wearable measurement system (Figure 1a). Thermal monitoring links vascular activity to skin-mediated thermal responses detected by a flexible array (Figure 1b), whereas arterial deformation provides the mechanical input for pulse and blood pressure sensing (Figure 1c). Skin surface electrical activity can be acquired through conformal ECG electrodes and compact readout electronics (Figure 1d), while respiratory monitoring may rely on abdominal deformation as a local mechanical proxy of breathing (Figure 1e).

Section 2, Section 3 and Section 4 apply this measurement sequence to thermal, mechanical, and skin surface electrical measurements, while Section 5 examines respiratory monitoring across several wearable sensing pathways. Each section begins by defining the physiological origin and the quantity available to the device, then examines how the interface and sensing process affect the recorded signal before considering the physiological information that can be supported. Section 6 compares the limitations shared across these sensing pathways and considers their implications for long-term use, multimodal systems, and clinical translation. Table 1 provides a cross-modal summary by linking direct measurands to supported outputs and the reference evidence required for stronger physiological or clinical claims.

Throughout this review, continuous monitoring describes the acquisition pattern within a defined recording session and does not by itself specify how long the device is worn. The terms extended and long-term are retained only when they describe evidence extending beyond a short experimental demonstration, but the actual on-body duration is reported whenever available rather than inferred from these labels. Long-term evidence may arise from sustained wear, repeated recording sessions, or follow-up measurements, and these study designs are not treated as equivalent. Acquisition continuity is reported separately from nominal wear duration, while effective valid data time excludes periods affected by signal loss or interruption. Mechanical cycling, environmental aging, and storage tests are treated as durability evidence rather than on-body monitoring evidence.

## 2. Flexible Temperature Sensors for Continuous Thermal-State Monitoring

Skin-mounted temperature sensors directly measure local skin temperature rather than core body temperature [41,42]. The surface temperature recorded by the device reflects heat delivered from deeper tissue together with heat exchanged with the surrounding environment, and the balance between these processes varies with local perfusion and anatomical location. The skin–sensor interface can further alter the recorded value through contact resistance, occlusion, and changes in surface heat loss. Local skin temperature can therefore support site-specific thermal assessment, whereas estimates of deeper or core temperature require an additional physiological or heat-transfer relationship that must be validated against an appropriate reference.

The main advantage of flexible temperature sensors lies in continuous and spatially resolved monitoring. Webb et al. developed an ultrathin conformal sensing-heating platform capable of mapping skin temperature and analyzing transient heat transport, thereby extending epidermal temperature sensing from single-point recording to localized thermal characterization [43]. Such measurements may support the identification of abnormal thermal regions and temporal trends, but local warming is not disease-specific and should be interpreted together with clinical or physiological information.

### 2.1. Physiological Meaning and Measurement Boundaries of Skin Temperature

Skin temperature reflects the balance between tissue heat delivery and environmental heat exchange [44]. Local blood flow contributes to this balance, but temperature and perfusion do not maintain a stable linear relationship because vascular responses exhibit delay, hysteresis, and site dependence [45,46]. A temperature change may therefore indicate altered peripheral heat regulation without providing a direct quantitative measure of blood flow.

Measurement location strongly affects both baseline temperature and environmental sensitivity. Distal regions are particularly susceptible to ambient temperature and vasoconstriction, whereas covered areas may be influenced by sweat accumulation and local occlusion. Longitudinal monitoring should maintain consistent placement, attachment conditions, and contact pressure, while recording relevant environmental and activity information. Repositioning the sensor may establish a new baseline and should not be interpreted automatically as a physiological change.

The temporal resolution of a temperature system is limited by the complete thermal pathway rather than by the electronic sampling rate alone. Heat capacity of the sensing layer, encapsulation thickness, and skin-sensor thermal resistance determine how rapidly the device approaches thermal equilibrium. Fever monitoring mainly requires stable low-frequency tracking, whereas transient thermal or perfusion studies require shorter thermal time constants and higher spatial resolution.

The sensor may also disturb the quantity being measured. Occlusive patches reduce evaporative and convective heat loss, while batteries, processors, or active heaters can introduce local heat sources or additional heat-dissipation paths. Evidence intended to support long-term thermal monitoring should demonstrate stable thermal contact and limited disturbance of the local skin microenvironment over the reported on-body period rather than infer long-term performance from sensor response or bench stability alone.

### 2.2. Resistive Temperature Sensors

Resistive temperature sensors convert temperature-dependent resistance changes into electrical output. Their practical accuracy depends on deformation sensitivity, humidity response, electrical noise, and long-term material stability; the key requirement is a reproducible resistance-temperature relationship under realistic wearing conditions.

#### 2.2.1. Metal Resistance Temperature Sensors

**Figure 2:**
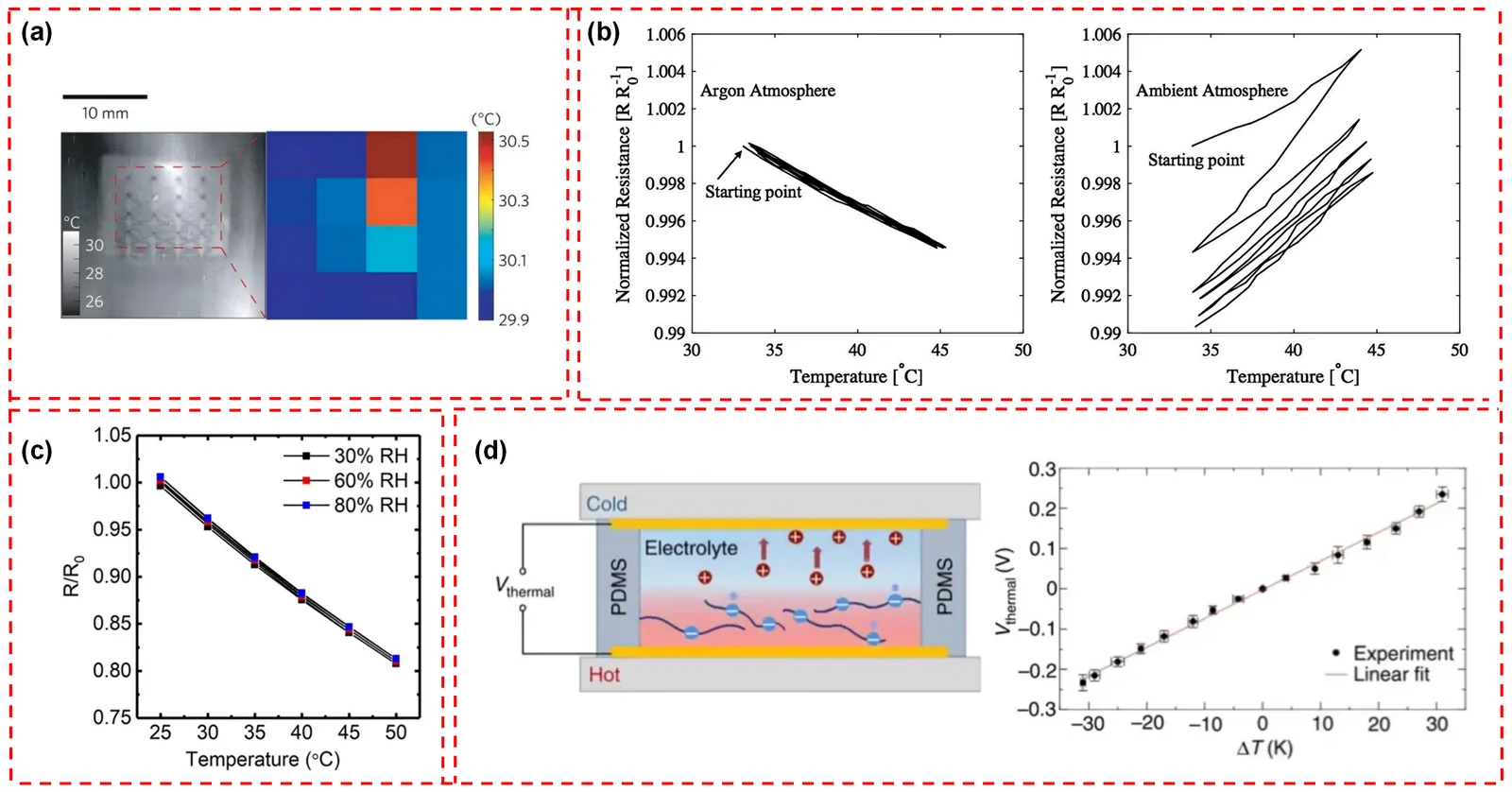
Environmental interference and transduction strategies in skin-interfaced flexible temperature sensors. (**a**) Ultrathin conformal temperature-sensor array mounted on the human wrist and the corresponding spatial map of local skin temperature, illustrating spatially resolved epidermal thermometry. Adapted with permission from Ref. [43]. (**b**) Temperature-dependent resistance responses of a graphene/PEDOT:PSS thermistor measured first in an inert atmosphere and subsequently in ambient air, showing substantial environmental cross-sensitivity in the absence of adequate moisture protection. Adapted from Ref. [51] under a Creative Commons Attribution 4.0 International License. (**c**) Temperature-dependent resistance responses of an optimized printed PEDOT:PSS thermistor at relative humidities of 30%, 60%, and 80%, demonstrating improved suppression of humidity-induced signal variation. Adapted from Ref. [52] under a Creative Commons Attribution 4.0 International License. (**d**) Schematic of ionic thermoelectric voltage generation through thermally induced ion redistribution and the corresponding linear relationship between thermal voltage and temperature difference. Adapted from Ref. [57] under a Creative Commons Attribution 4.0 International License.

Metal resistors provide comparatively stable and calibratable temperature responses. Because thin metallic films have limited intrinsic stretchability, mechanical compliance is generally achieved through serpentine, mesh, or wavy layouts that convert macroscopic stretching into local bending [47,48,49,50]. Reducing device thickness further improves conformity to skin microtexture and lowers thermal mass. In the platform developed by Webb et al., metallic resistance elements and microscale heaters were integrated into an ultrathin structure for local temperature mapping and transient thermal analysis, with the conformal array resolving spatial differences in wrist temperature (Figure 2a) [43].

Geometric compliance does not completely eliminate deformation-induced resistance changes. Motion may still alter the conductive path or concentrate stress near interconnections, producing temperature-equivalent noise. Sensitive elements should therefore be positioned in low-strain regions and combined with differential or reference measurements when dynamic monitoring is required.

Because metallic resistance changes are modest, lead resistance, conversion noise, and Joule self-heating should be controlled through low-current excitation, compensation, and stable references.

#### 2.2.2. Composite Thermistors

Composite thermistors amplify temperature response through thermally activated transport or reconstruction of conductive networks. Printing and coating methods allow these materials to be deposited on soft substrates, but their output is sensitive to composition, filler dispersion, film formation, and residual solvent. Vuorinen et al. printed graphene/PEDOT thermistors on a skin-conformable polyurethane substrate, demonstrating scalable fabrication while also revealing substantial environmental sensitivity, as reflected by the different resistance-temperature responses measured in inert and ambient conditions (Figure 2b) [51].

Hydrophilic conductive composites are particularly vulnerable to humidity interference because absorbed water changes both polymer structure and charge transport. Wang et al. reduced this effect by cross-linking PEDOT and introducing a CYTOP passivation layer, enabling a more consistent temperature response across relative humidities from 30% to 80% (Figure 2c) [52]. The added barrier, however, also alters thermal transport and may slow the response, so humidity protection should be optimized together with thermal time constant and skin permeability.

High sensitivity is often achieved near the percolation threshold, where small structural changes strongly affect electrical conduction. This condition also increases susceptibility to mechanical cycling and irreversible network rearrangement. Hao et al. used a nacre-inspired laminated MXene structure to dissipate in-plane strain before it reached the thermosensitive conductive network, thereby reducing strain interference without relying entirely on post-processing algorithms [53].

### 2.3. Transistor, Thermoelectric, and Heat-Flux-Based Strategies

Transistor temperature sensors use temperature-dependent channel transport or interface charge to generate an amplified current response [54,55,56]. Their local gain is advantageous for active arrays and small temperature differences, but channel current also changes with bias instability, trap states, semiconductor aging, and mechanical deformation. A high current gain therefore improves readability without necessarily reducing absolute temperature error.

Zhao et al. developed ionic thermoelectric-gated organic transistors in which a temperature gradient drives ion redistribution and modulates the transistor channel, with the resulting thermovoltage varying with the applied temperature difference (Figure 2d) [57,58]. This architecture amplifies weak temperature differences, although the gain remains dependent on electrolyte water content, ion migration, encapsulation, and long-term baseline stability.

Thermocouples and thermopiles generate voltage from a temperature difference and avoid continuous electrical excitation of the sensing junction. Their output represents a relative temperature gradient rather than absolute skin temperature, so a stable reference junction is required. Changes in clothing, airflow, or environmental temperature may alter the reference condition and must be considered during calibration.

Zero-heat-flux sensors use heating and thermal insulation to reduce outward heat loss until local skin temperature approaches that of deeper tissue. This method provides a more explicit basis for core-temperature estimation than ordinary surface thermistors, but it requires longer stabilization and remains sensitive to tissue thickness and sensing location. Validation should therefore use a core-temperature reference appropriate to the intended clinical setting.

Pyroelectric materials generate charge mainly during temperature change and lose output under steady conditions. They are more suitable for detecting transient thermal events or rates of change than for continuous absolute-temperature measurement. When combined with resistive or thermoelectric channels, they may provide complementary dynamic information but should not replace a calibrated static-temperature channel.

### 2.4. Structural and Interfacial Design

The primary structural requirement for skin temperature sensing is stable thermal coupling. Thick or rigid devices leave microscopic air gaps that increase contact thermal resistance and delay equilibration [59,60,61]. Ultrathin, low-modulus architectures improve conformity and reduce thermal mass, but they also become more susceptible to wrinkling, stretching, and interconnection-induced stress. Thermal and mechanical pathways should therefore be designed together so that heat reaches the sensing layer efficiently while body motion is diverted away from the temperature-sensitive element.

Encapsulation must balance environmental isolation with preservation of the skin microenvironment. Impermeable layers protect the sensor from sweat but may trap heat and moisture, whereas porous or semipermeable structures improve breathability while increasing exposure to environmental water vapor and airflow [62,63]. Chen et al. integrated serpentine metallic temperature sensors with a membrane that permitted gas and water-vapor transport while blocking liquid water, demonstrating a practical approach to breathable encapsulation [64]. Such packaging improves wearing conditions but does not inherently remove strain interference from the resistive sensing channel.

Temperature arrays reduce dependence on a single contact point and enable spatial comparison of thermal patterns. Their interpretation requires pixel-to-pixel consistency and uniform thermal contact because nonuniform encapsulation, wiring, or local electronics may generate apparent spatial differences unrelated to tissue state. Calibration should therefore account for both individual sensing units and the thermal behavior of the complete array.

Closed-loop thermal systems introduce an additional challenge because the actuator changes the quantity being measured. Huang et al. integrated an MXene temperature sensor with a thermoelectric cooling module and feedback electronics to demonstrate local closed-loop control [65]. The sensing element and reference channel must be positioned so that physiological temperature changes can be separated from actuator-generated heat; otherwise, the feedback system may respond primarily to its own thermal disturbance.

Taken together, these examples show that accurate skin thermometry requires more than high temperature sensitivity because spatial heterogeneity, environmental humidity, and the transduction mechanism can all affect the recorded signal.

### 2.5. Performance Evaluation and Remaining Challenges

Evaluation should begin by defining the intended measurand. Local skin temperature can be compared with a calibrated surface reference, spatial imaging requires assessment of pixel consistency, and core-temperature estimation requires synchronization with an appropriate core reference. A reproducible warming-cooling response supports thermal trend tracking, whereas absolute or core-temperature claims require reference-based validation.

Response time should be evaluated under thermal boundary conditions that resemble actual use, because the behavior of an encapsulated skin-mounted device differs from that of a bare sensing film under ideal laboratory switching [66,67]. Measurement reliability should therefore be assessed through accuracy and temporal stability rather than isolated performance metrics.

Cross-sensitivity should be assessed under combined temperature, deformation, and humidity changes rather than through isolated tests. Claims of strain insensitivity or humidity stability should be validated under application-relevant mechanical and environmental conditions with clearly defined testing duration. Dynamic human studies should also report environmental conditions, sweating state, attachment method, and any recalibration required after repositioning.

Studies intended to support extended or long-term thermal use should report the actual on-body duration and acquisition pattern before assessing thermal-contact drift, adhesion loss, repositioning, invalid data intervals, or recalibration during that period. Bench cycling and environmental aging should be reported separately because they establish device durability rather than on-body monitoring duration.

The suitability of a flexible temperature sensor is therefore determined by more than its nominal temperature sensitivity. A useful system must preserve a reproducible relationship between local tissue heat transfer and the recorded temperature while limiting changes introduced by the interface, environment, and device itself [60,68,69]. The strength of any subsequent physiological interpretation depends on whether that relationship remains stable under the intended wearing conditions and has been tested against a reference appropriate to the claimed thermal quantity. When skin temperature is reported as an absolute quantitative output, comparison with an appropriate reference should characterize measurement error and agreement across the intended temperature and environmental range rather than rely on correlation alone.

## 3. Flexible Mechanical Sensors for Pressure, Strain, and Pulse Monitoring

Physiological motion reaches the skin as deformation whose amplitude, direction, and frequency depend on both its source and the intervening tissue. Arterial pulsation generates relatively small surface motion, whereas joint movement and muscle contraction produce larger and more complex deformation [70,71]. Before this mechanical input reaches a flexible sensor, tissue properties, anatomical location, placement, preload, and attachment can modify both its magnitude and waveform [72,73,74]. The resulting electrical output therefore reflects the combined effects of physiological motion, mechanical transmission, and sensor response. A clear waveform alone cannot establish that the intended mechanical quantity has been isolated, particularly when multiple forms of loading occur simultaneously during wear.

### 3.1. Physiological Signals and Sensing Requirements

Arterial pulse waves originate from ventricular ejection and produce periodic displacement after propagation through vessels and superficial tissue. Sensors positioned above the radial or carotid artery can record heartbeat-related waveforms whose timing and morphology contain information related to vascular motion. The surface waveform is a tissue-filtered proxy that can support heart-rate and waveform-trend analysis, whereas hemodynamic metrics require controlled coupling and independent validation.

Joint and muscle monitoring involves larger deformation and therefore requires a wider working range, directional selectivity, and stable cycling performance. Joint flexion simultaneously stretches, bends, and compresses the skin, while muscle contraction changes both surface curvature and tissue stiffness. Sensors used in these applications should maintain repeatable output throughout the relevant deformation range rather than achieve a high gauge factor only near structural failure.

Thoracoabdominal motion represents a low-frequency mechanical input strongly affected by posture and material viscoelasticity. Local strain can identify respiratory cycles, but its quantitative relationship with tidal volume is not universal. The respiratory application of strain and pressure sensors is therefore discussed in Section 5.

Heart sounds, respiratory sounds, and laryngeal activity occupy higher frequency ranges and often overlap in space and time. Their acquisition depends on anatomical placement, skin coupling, bandwidth, and signal separation. Lee et al. developed a soft wireless mechano-acoustic system capable of recording cardiopulmonary and laryngeal vibration for respiratory classification [75]. This work illustrates that weak physiological vibration can be captured by soft devices, although the resulting information remains sensitive to placement and algorithmic processing.

Accordingly, pulse, joint-motion, and mechano-acoustic sensors should be evaluated against modality-specific requirements for waveform fidelity, working range, bandwidth, and noise.

### 3.2. Piezoresistive Sensors

Piezoresistive sensors convert deformation into resistance variation through changes in conductive pathways, particle contact, tunneling distance, or electrode-material interfaces [76,77]. Their simple readout and compatibility with printable composites make them widely used for pressure and strain sensing, but the conductive network may also respond to temperature, humidity, viscoelastic relaxation, and irreversible structural rearrangement.

Networks close to the percolation threshold generate large resistance changes under small deformation, thereby increasing sensitivity while also increasing baseline drift, device variability, and dependence on loading history [78,79,80,81]. Increasing the conductive fraction can improve stability but usually raises stiffness and reduces relative response. Accordingly, network composition should be optimized for calibration stability within the target physiological range.

Microstructures amplify weak loads by concentrating deformation and controlling contact-area evolution. Pan et al. used a hollow-sphere conducting-polymer structure to achieve pressure detection below 1 Pa, demonstrating that low-pressure response can be governed by structural compressibility rather than material conductivity alone [82,83]. Porous, protruding, crack-based, and wrinkled structures follow the same general principle, but their benefits are accompanied by nonlinearity, hysteresis, saturation, or limited range.

Piezoresistive calibration is affected by polymer creep, loading rate, temperature, humidity, and interfacial fatigue. These effects should be quantified within the mechanical range and frequency of the intended physiological signal.

### 3.3. Capacitive and Iontronic Sensors

Capacitive sensors use deformation-induced changes in electrode spacing, overlap area, or dielectric properties [84,85,86,87]. Because they do not depend on reconstruction of a conductive percolation network, they often provide good cycling repeatability, although their small signals are susceptible to parasitic capacitance, electromagnetic coupling, and interconnection motion.

Weak-pressure sensitivity is commonly improved by introducing porous or surface-microstructured dielectric layers that reduce effective stiffness and concentrate deformation [88,89,90,91]. Changes in dielectric thickness and effective permittivity may jointly amplify capacitance, but progressive densification eventually causes nonlinear response or saturation. High-permittivity fillers increase the initial capacitance but may also increase stiffness and dielectric loss, so their benefits must be evaluated together with compressibility and frequency response.

**Figure 3:**
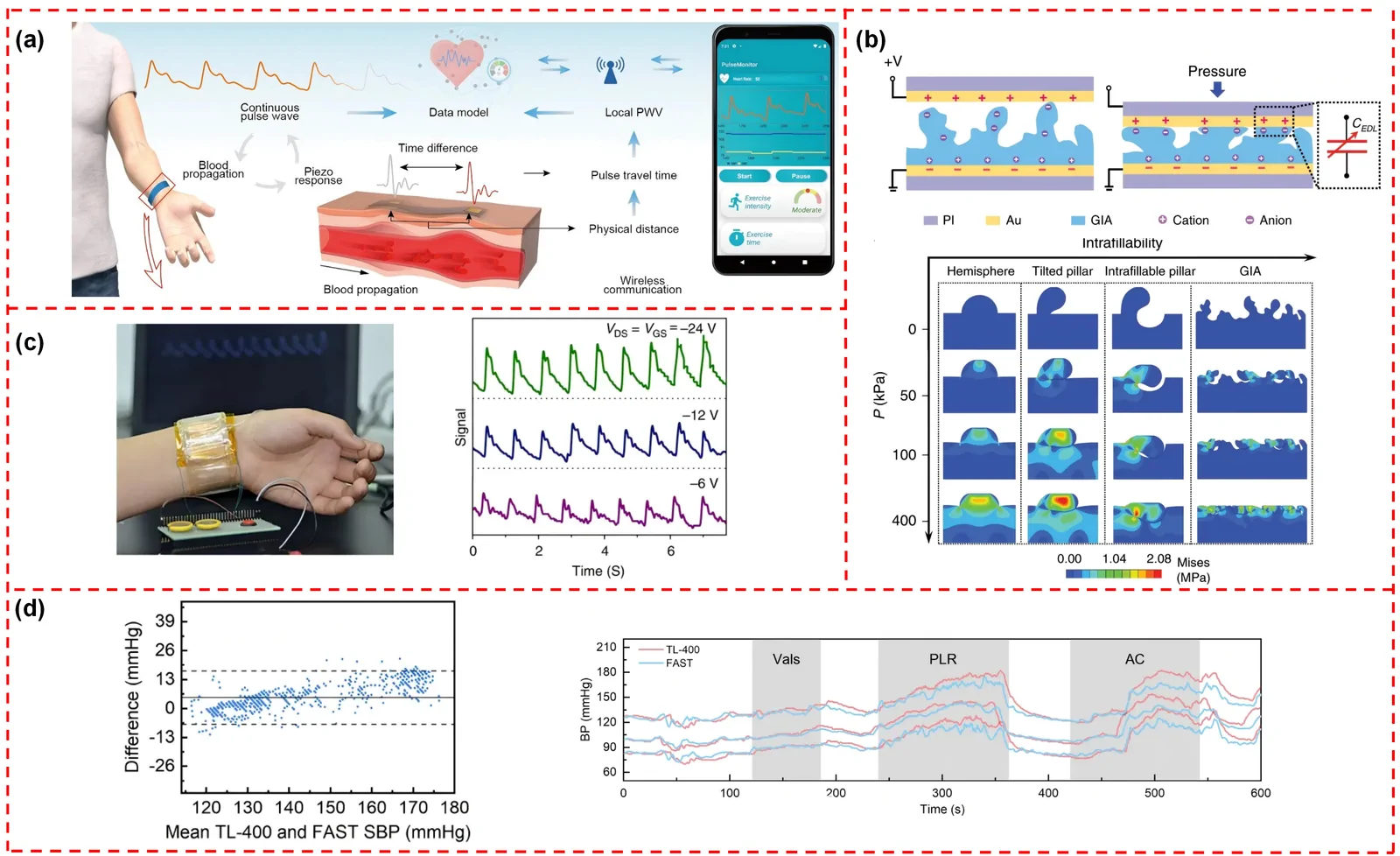
Flexible mechanical sensing from body surface deformation to physiological validation. (**a**) Exploded architecture and on-wrist configuration of a thin, soft piezoelectric sensing system for continuous arterial pulse and blood pressure monitoring, illustrating the integration of conformal sensing, active pressure adaptation, and wireless readout. Adapted with permission from Ref. [15]. (**b**) Finite-element comparison of stress distributions in representative surface architectures and the operating principle of a graded intrafillable iontronic pressure sensor before and after pressure application. Progressive structural filling and contact-area evolution increase electric-double-layer capacitance over a broad pressure range. Adapted from Ref. [92]. (**c**) Battery-powered flexible suspended-gate organic thin-film transistor attached over the radial artery and the corresponding real-time pulse-wave signals under different operating voltages, demonstrating on-body acquisition of weak arterial pressure signals. Adapted from Ref. [98]. (**d**) Continuous systolic, mean, and diastolic blood pressure measurements obtained using flexible adaptive sensing tonometry and a medical-grade TL-400 reference during Valsalva breathing, passive leg raising, and air cycling, together with Bland–Altman analysis of systolic blood pressure. Adapted from Ref. [99].

Iontronic sensors amplify pressure-induced contact changes through electric double-layer capacitance. Bai et al. introduced a graded intrafillable structure that maintained contact-area evolution over a broad pressure range, thereby combining high low-pressure response with an extended operating range (Figure 3b) [92]. The gain nevertheless depends on ion migration, water content, temperature, and measurement frequency, making encapsulation and dynamic calibration essential.

Capacitive readout requires low-noise electronics and control of parasitic coupling; shielding and nearby circuits may improve signal quality while increasing local stiffness.

### 3.4. Dynamic and Transistor-Based Sensors

Piezoelectric materials generate charge under changing mechanical stress and are therefore suitable for pulse, heart sounds, respiratory vibration, and impact [93]. Because the charge dissipates under constant load, these devices are less suitable for sustained static-pressure measurement. Their waveform also depends on device capacitance and readout impedance, so performance should be evaluated as a sensor-circuit system.

Triboelectric sensors convert contact and separation into electrical signals through contact electrification and electrostatic induction. Their high output voltage and self-powered operation support rhythm and event detection, but the response is strongly affected by contact speed, surface condition, humidity, and preload [94,95,96]. Sweat accelerates charge dissipation and repeated friction changes the interface, limiting their use for quantitative pressure measurement.

Transistor-based sensors integrate mechanical sensing and local amplification. Schwartz et al. combined a microstructured dielectric with a polymer transistor to amplify pressure-induced capacitance changes and record radial pulse signals [97]. Zang et al. used a suspended-gate structure and air gap to detect pressures below 0.5 Pa and record acoustic waveforms [98]. The same suspended-gate platform also enabled on-body acquisition of radial pulse waveforms under different operating voltages (Figure 3c), illustrating the use of local transistor amplification for weak arterial signals. Transistor gain increases dependence on bias stability, semiconductor aging, structural consistency, and circuit integration, limiting its advantage to applications that require local amplification or active-matrix addressing. At the system level, conformal piezoelectric sensing can be integrated with active pressure adaptation and wireless readout to support continuous arterial monitoring (Figure 3a). Together with the graded iontronic architecture discussed above (Figure 3b), these examples show that structural and transistor-based strategies can improve the detectability of weak mechanical inputs. Such improvements in signal acquisition do not by themselves establish physiological validity, which requires preservation of waveform fidelity and comparison with an appropriate reference under relevant physiological variations.

### 3.5. Pulse Waveform Fidelity and Blood Pressure Interpretation

Although these architectures improve mechanical detectability, they do not by themselves establish cardiovascular validity. Cardiovascular interpretation therefore requires preservation of pulse timing and morphology. Limited bandwidth, viscoelastic damping, resonance, and excessive filtering can distort reflected-wave and propagation features [22,100,101].

Contact pressure and positioning are major sources of variation. Misalignment increases tissue attenuation, insufficient preload weakens coupling, and excessive pressure alters vascular motion. Kaisti et al. used a wearable MEMS pressure array to record radial pulse waveforms and evaluate heart rate and atrial fibrillation, showing that arrays reduce misalignment risk but do not eliminate the need for stable wrist coupling [99,102]. Posture, wrist height, and tissue thickness should also be controlled or recorded during longitudinal monitoring.

Estimating blood pressure from surface pulse waves requires substantially stronger evidence. Pulse amplitude and morphology are influenced by vascular tone, sensing location, tissue coupling, and individual calibration, preventing a universal conversion to systolic or diastolic pressure [103,104,105]. Machine-learning models must therefore be validated in independent participants and during actual blood pressure changes [106,107].

ISO 81060-3:2022 [21] specifies clinical investigation procedures for continuous automated non-invasive sphygmomanometers and distinguishes between trend-monitoring and absolute-accuracy claims. A flexible pulse sensor reporting blood pressure must consequently be evaluated beyond waveform correlation or comparison with a single cuff measurement. Quantitative blood pressure claims should report measurement bias and agreement across the tested pressure range rather than waveform correlation alone. The comparison of continuous systolic, mean, and diastolic measurements with a medical-grade reference during controlled physiological perturbations in Figure 3d illustrates the type of evidence required for such quantitative claims. When repeated observations are obtained from the same participants, the analysis should account for the within-subject data structure rather than treat all measurement pairs as independent.

Recent epidermal systems also show that continuous blood pressure monitoring is becoming a system-level problem rather than a sensor-only problem. Kurt et al. developed a battery-free epidermal sensor network in which wireless power delivery and data communication were separated through a wearable metamaterial textile, enabling continuous systolic blood pressure monitoring including during exercise [3]. This architecture reduces the burden of a local battery but introduces additional requirements for power-transfer stability, communication continuity, and system-level validation during physiological measurement.

Cross-modal acquisition provides another route to cardiovascular measurement when the additional signal carries information that is not available from a single mechanical channel. Wu et al. integrated biopotential and biomechanical responses within one epidermal sensor and used the resulting cross-modal signal to derive heart rate, pulse-arrival time, and blood pressure outputs [4]. Such integration can reduce device footprint and channel count, but the physiological contribution of each signal component and the validity of the derived output still require explicit separation and reference-based evaluation.

### 3.6. Performance Evaluation and Research Priorities

Mechanical sensor evaluation should be matched to the intended physiological signal and should consider both the sensing range and dynamic loading conditions to ensure meaningful comparison across studies. Cross-study sensitivity values are interpretable only when these conditions are comparable.

Combined loading should be assessed because body-worn sensors rarely experience a single stimulus. Structural isolation, reference channels, and controlled attachment should be used to reduce cross-sensitivity before physiological waveform analysis. Recent soft ionic sensing architectures have also demonstrated intrinsic separation of force and temperature responses through device-level transduction design [5], illustrating a route to reduce cross-sensitivity before downstream signal processing. Such demonstrations establish a decoupling strategy but do not by themselves validate a physiological output. Claims involving extended or long-term use should be supported by reported on-body recording periods under realistic wearing conditions, with signal stability and interface reliability evaluated across those periods. Repeated loading or deformation cycles should be reported as mechanical durability and should not be used as evidence of continuous or long-term physiological monitoring.

Mechanical amplification can improve the detectability of weak physiological motion, but this advantage is meaningful only when the recorded waveform remains reproducibly related to the underlying event. Changes in coupling or unintended loading can alter that relationship even when the sensor itself retains high sensitivity. Mechanical sensor performance should therefore be judged by the fidelity and reproducibility of the physiological information obtained under the intended wearing conditions, with stronger derived claims supported by an appropriate reference measurement.

## 4. Flexible Electrodes for Surface Biopotential and Electrodermal Monitoring

Skin surface electrical measurements depend on the transfer of ionic physiological signals across a variable electrode interface into an electronic recording system. Biopotentials arise from electrical activity in excitable tissues, whereas electrodermal activity reflects changes in skin conductance associated primarily with sweat-gland activity. Their physiological origins differ, but both are strongly affected by the condition of the skin-electrode contact before the signal reaches the recording circuit. Variations in hydration, contact area, relative motion, and electrode placement can therefore change the measured signal without a corresponding change in the underlying physiological process.

### 4.1. Signal Characteristics and Measurement Boundaries

Electrocardiography (ECG) records the potential distribution produced by cardiac electrical activation [108,109,110]. Flexible electrodes can reduce wearing burden and extend monitoring duration, but they do not alter the physiological meaning or diagnostic requirements of the ECG waveform. Lead position, analog front-end characteristics, filtering, and sampling conditions must remain controlled before a flexible system can be compared with clinical instrumentation.

Surface electromyography (sEMG) represents the summation of motor-unit action potentials detected at the skin surface [111,112,113]. Its amplitude and spectral characteristics depend strongly on electrode position, interelectrode spacing, muscle-fiber orientation, and crosstalk from adjacent muscles. Repositioning the electrode may therefore alter the signal independently of muscle function. Longitudinal or rehabilitation applications require reproducible placement and should not attribute all between-session variation to physiological change.

**Figure 4:**
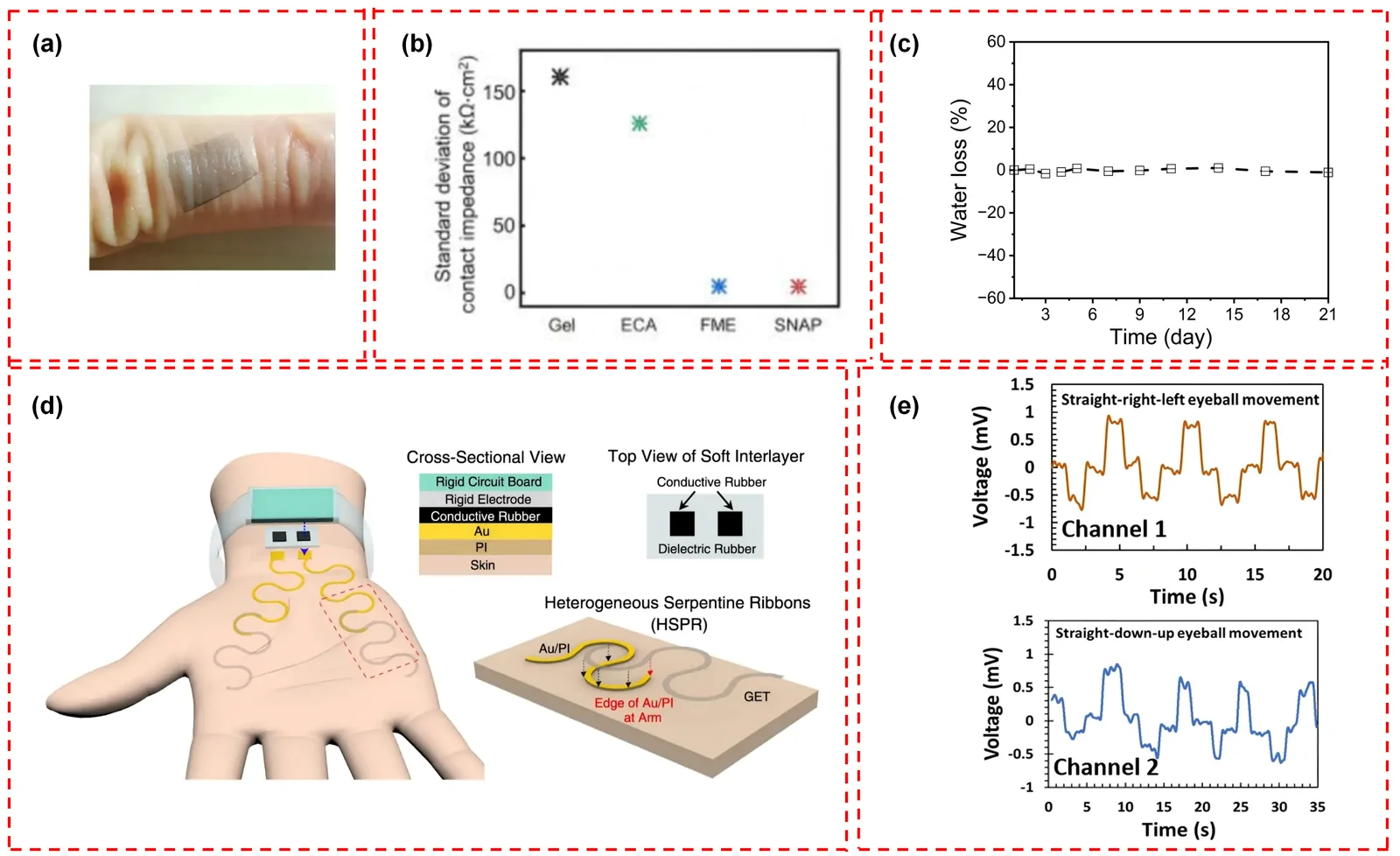
Interface stabilization and anatomical adaptation for skin biopotential and electrodermal sensing. (**a**) ECG signals recorded using ultrathin PEDOT:PSS/graphene, PEDOT:PSS, and conventional Ag/AgCl electrodes under static and vibration conditions, illustrating the suppression of motion-induced signal distortion by an ultraconformal dry interface. Adapted from Ref. [122], with permission. (**b**) Variability of skin-contact impedance for gel, conductive-adhesive, flexible microneedle, and stretchable microneedle adhesive electrodes under sweat, dead-skin-cell, hair, and contamination conditions, demonstrating the reduced dependence of microneedle interfaces on variable skin surface states. Adapted from Ref [120], with permission. (**c**) Twenty-one-day anti-drying testing of an approximately 10 μm thick nanomesh-reinforced hydrogel, demonstrating material-level hydration stability rather than 21-day on-body electrophysiological recording. Adapted from Ref. [121], with permission. (**d**) System architecture of a palm-mounted graphene electronic tattoo connected to a wrist-mounted EDA acquisition unit through heterogeneous serpentine interconnects and a soft conductive interlayer, illustrating the integration of a physiologically responsive measurement site with rigid acquisition electronics. Adapted from Ref. [28], with permission. (**e**) Horizontal and vertical EOG signals recorded using ultrathin graphene electronic-tattoo electrodes during left-right and up-down eye movements, demonstrating conformal biopotential acquisition from the curved and highly mobile periocular region. Adapted from Ref. [117], with permission.

Electrooculography (EOG) measures eye movement through the corneo-retinal standing potential [114,115,116]. The curved and highly mobile periocular region creates demanding interface conditions, while blinking and facial muscle activity partially overlap with the target signal. Ultrathin electrodes may improve conformal contact, as demonstrated by graphene electronic-tattoo electrodes that record horizontal and vertical EOG during left-right and up-down eye movements (Figure 4e) [117]. Rigid connectors or poorly designed interconnections can still pull the skin and introduce motion artifacts.

Electrodermal activity (EDA) is usually obtained by applying a small voltage or current and measuring time-dependent skin conductance. The signal is commonly separated into a slowly varying tonic component, often expressed as skin conductance level (SCL), and faster phasic skin conductance responses (SCRs) [38,118]. These components represent different temporal processes and require defined measurement modes, response windows, and decomposition methods. A single average conductance value does not preserve the full physiological information.

### 4.2. Skin-Electrode Interface

The skin–electrode interface combines ionic conduction in tissue, electronic conduction in the electrode, and interfacial polarization. The stratum corneum is the dominant electrical barrier, and its hydration, integrity, and thickness vary across body sites and over time. Interface impedance is frequency-dependent, and measurements obtained under a single condition may not fully represent signal coupling during practical physiological recording.

Conventional wet Ag/AgCl electrodes reduce impedance by hydrating the stratum corneum and increasing effective contact area through an electrolyte gel. This provides a stable initial interface but introduces time-dependent changes as the gel dries or the occluded skin becomes more hydrated. Wet electrodes are therefore reliable for many short-term recordings, whereas prolonged use may be limited by baseline drift, skin irritation, and declining adhesion.

Dry electrodes avoid pre-applied gel and rely on direct conformal contact. Their performance is more sensitive to skin microtexture, hair, wrinkles, and motion because small changes in contact area can alter both impedance and interfacial potential. These artifacts often overlap with low-frequency physiological components and cannot be removed completely by filtering. Zhang et al. developed a self-adhesive, fully organic dry electrode that combined conductivity, stretchability, and skin adhesion, enabling ECG and EMG recording during sweating and body motion with lower dynamic noise than conventional gel electrodes [119].

Contact pressure can reduce the interface gap, but it cannot be increased indefinitely. Moderate pressure helps to reduce impedance, while excessive pressure may cause discomfort and change superficial tissue morphology and local sweating state. The contact pressure of chest strap or fabric electrode will also change with posture and clothing tightness, so that the low impedance at rest can not represent the actual performance in daily activities. Reasonable flexible electrodes should rely on structural compliance and moderate adhesion to maintain the interface, rather than continuous external compression.

### 4.3. Interface Strategies for Biopotential Recording

The following electrode types are best understood as interface-stabilization strategies rather than mutually exclusive material categories. Electrolyte gels, hydrogel networks, direct dry contact, ultrathin structures, and microneedles may be combined within the same device. Their main distinction lies in how they maintain ionic coupling, reduce relative motion, and accommodate the intended wearing duration.

#### 4.3.1. Wet and Dry Interfaces

Flexible wet electrodes retain the mature Ag/AgCl–electrolyte interface while reducing substrate thickness and mechanical mismatch. Their initial impedance is generally favorable, but their long-term stability remains limited by water loss, gel redistribution, and occlusion of the skin. Flexible construction improves mechanical comfort without eliminating the aging mechanism of the wet interface.

Dry electrodes may begin with higher impedance but can provide more stable mechanical contact during prolonged or repeated use. Their effectiveness depends on low-modulus substrates, deformable conductive layers, and surface structures that follow skin microtexture. Meshes and serpentine interconnections reduce strain in conductive regions, while microstructured or adhesive surfaces increase real contact area.

Comparisons between wet and dry electrodes should be tied to a reported on-body monitoring period rather than initial impedance alone. Time-dependent interface changes and signal failure should be interpreted in relation to the duration and acquisition pattern of the recording, while repeated removal or reattachment should be reported separately because these procedures introduce additional interface changes.

#### 4.3.2. Microneedle Interfaces

Microneedle electrodes partly bypass the high-impedance stratum corneum and can improve recording in regions where planar contact is unstable. Kim et al. developed a stretchable microneedle adhesive patch that acquired electrophysiological signals without prior skin preparation and maintained contact during movement [120]. Compared with planar gel and conductive-adhesive interfaces, the microneedle configurations showed less variation in skin-contact impedance under sweat, dead-skin-cell, hair, and contamination conditions (Figure 4b). The flexible backing reduced mechanical mismatch, while the microneedles provided a low-impedance pathway.

This gain is achieved through slight penetration of the skin barrier, so microneedle electrodes should not be compared with non-invasive planar electrodes solely by impedance. Safety, discomfort, needle integrity, repeated use, and the effect on the skin barrier must also be considered.

#### 4.3.3. Hydrogel and Ionically Conductive Interfaces

Hydrogels provide a soft, water-rich network that conforms to skin microtexture and supports ionic conduction between tissue and the electronic electrode. Their low modulus and high-water content can reduce contact impedance, while incorporation of an electronic conductive phase shortens the conversion pathway from ionic to electronic current.

The same water content that improves coupling also creates the principal stability problem. Dehydration increases stiffness and reduces ionic conductivity, whereas excessive swelling changes geometry and adhesion. Humectants and barrier layers can slow water loss but may alter permeability, biocompatibility, or thermal transport. Hydrogel electrodes should therefore be evaluated through time-dependent mass, mechanical, and impedance changes rather than initial conductivity alone.

Zhang et al. reported a gas-permeable hydrogel sensor reinforced by a nanomesh approximately 10 μm thick [121]. The 21-day anti-drying test demonstrates material-level hydration stability (Figure 4c), while the nanomesh provides mechanical support during handling and wear. This material test should not be interpreted as evidence of 21 days of continuous on-body electrophysiological recording.

#### 4.3.4. Ultrathin Epidermal Electrodes and Electronic Tattoos

Ultrathin electrodes reduce bending stiffness and follow skin microtexture with minimal mechanical loading. Their main benefit is improved interface stability rather than an increase in intrinsic electrical sensitivity. When combined with appropriate adhesion, low bending stiffness limits relative sliding and reduces motion-induced impedance changes. This advantage is reflected in ECG recordings under vibration, where an ultraconformal PEDOT:PSS/graphene interface suppresses motion-induced waveform distortion relative to less conformal electrode configurations (Figure 4a).

Ultrathin construction also decreases handling tolerance. Films may wrinkle or tear during transfer and removal, and rigid connectors can concentrate stress at the electrode edge. The complete mechanical design is therefore often limited by interconnections rather than by the sensing film itself.

Electronic tattoos address part of this problem by placing thin conductive patterns directly on the skin. Jang et al. used graphene electronic tattoos on the palm and heterogeneous serpentine interconnections to maintain electrical continuity between the ultrathin electrode and the wrist-mounted circuit during free movement [28]. The study also demonstrated that anatomical location and interconnection design jointly determine whether a physiologically responsive site can be used in ambulatory monitoring.

### 4.4. Electrodermal Activity Monitoring

EDA is sensitive to sympathetic arousal but is not specific to a particular emotion or disease. Psychological stress, pain, exercise, heat exposure, and other stimuli may all increase skin conductance. EDA should therefore be interpreted as an index of physiological activation within a defined experimental context rather than as an independent identifier of emotional state.

Measurement location strongly influences EDA amplitude and dynamics because sweat-gland density and skin properties differ across the body. Palmar and plantar sites generally provide strong responses but may be inconvenient for daily wear, whereas wrist locations are easier to integrate but often produce weaker or differently shaped signals. Thresholds and models developed at one site should not be transferred directly to another without validation.

The graphene electronic-tattoo system reported by Jang et al. enabled unobtrusive palmar EDA recording during free movement by connecting the high-response palm location to a wrist-mounted electronic module (Figure 4d) [28]. This design illustrates that electrode placement determines physiological signal strength, while mechanical interconnection determines practical usability.

Multimodal information may help restrict EDA interpretation when it provides independent context. Activity and temperature channels can indicate whether conductance changes coincide with movement or thermal stress, while cardiopulmonary signals may provide complementary autonomic information. Under heavy sweating, however, interfacial water accumulation may drive conductance toward saturation and reduce the visibility of subsequent responses. EDA systems validated during resting psychological tasks should therefore not be assumed to retain the same performance during exercise. Across biopotential and electrodermal modalities, electrode performance is determined not by conductivity alone, but by how the interface accommodates skin microtexture, surface contamination, hydration changes, anatomical curvature, and mechanical connection to the acquisition electronics.

### 4.5. Signal Quality During Prolonged and Repeated Recording

Although the strategies summarized in Figure 4 can improve interfacial stability, their practical value must ultimately be evaluated within the complete recording chain. Material integrity, interface impedance, analog front-end characteristics, and modality-specific signal fidelity jointly determine waveform quality.

Static recordings establish only basic acquisition capability. Zhang et al. compared their self-adhesive dry electrodes with standard Ag/AgCl electrodes under dry, sweaty, and moving conditions and further demonstrated recording in participants with atrial fibrillation [119]. Such dynamic and target-population tests provide more meaningful evidence than a short, filtered waveform acquired from a stationary healthy participant.

Electrical signal validation should be tied to the physiological information extracted from the waveform rather than to a single signal-to-noise ratio. Measurements used for timing or morphology should quantify the corresponding error against a synchronized reference, whereas event or classification outputs require event-level or class-specific accuracy. Signal quality metrics remain useful for identifying degraded recordings but do not substitute for validation of the physiological output derived from those recordings.

Motion artifacts may arise from electrode sliding, contact-area changes, or deformation-induced interfacial potentials. Electrode detachment and abrupt contact changes should be classified as invalid recording intervals, while stable adhesion and low-traction interconnections should be established before signal processing.

Electrode studies intended to support long-term use should report the duration of each on-body recording session and state whether acquisition was continuous, intermittent, or repeated across separate sessions. Signal loss and skin reactions should be linked to these recording periods, while removal and reattachment should be analyzed separately because they test different interface failure modes. Validation should include the intended user population because electrical coupling and skin tolerance may differ from those observed in short-duration studies of healthy participants.

### 4.6. Section Summary

The comparisons in this section show that electrode performance is governed by the stability of the physiological signal as it crosses the skin–electrode interface rather than by conductivity or initial impedance alone. Strategies that improve contact can reduce one source of variability while introducing others related to hydration, mechanical loading, durability, or skin tolerance. Their value should therefore be assessed through the quality and reproducibility of the recorded physiological signal over the intended wearing period. This perspective also places interconnection design and front-end electronics within the same measurement problem because disturbances introduced after the electrode can compromise information that was preserved at the skin interface.

## 5. Flexible Sensors for Respiratory Monitoring Across Multiple Sensing Pathways

Respiratory monitoring illustrates the difficulty of interpreting a physiological process from a wearable-accessible proxy because the device generally does not measure ventilation directly. Respiratory activity produces several measurable changes at or near the body surface, and each retains only part of the information contained in the underlying respiratory process. The physiological meaning of the recorded signal therefore depends on how closely the selected proxy is related to the intended output and how strongly that relationship changes with placement, posture, motion, and individual anatomy. This section evaluates respiratory sensors on that basis rather than assuming that detection of a periodic breathing-related waveform is sufficient to establish a quantitative respiratory measurement.

### 5.1. Respiratory Parameters and Their Measurement Boundaries

Respiratory rate is usually derived from the timing of repeated cycles and is therefore less dependent on absolute signal amplitude than respiratory volume. Under resting conditions, a stable periodic waveform may provide reliable rate estimates. Shallow or irregular breathing, however, reduces the clarity of cycle boundaries, while body motion may generate signals in a similar frequency range and cause missed or duplicated events. Performance under controlled resting respiration cannot therefore establish reliability during sleep or daily activity.

Respiratory depth and tidal volume require a quantitative relationship between the measured surface signal and actual airflow or gas volume. Thoracic and abdominal deformation varies with posture, breathing pattern, sensor location, and individual anatomy, so identical tidal volumes may produce different local strain amplitudes. Any system reporting tidal volume from body surface deformation should be calibrated against a flow- or volume-based reference and re-evaluated when posture or activity changes. Wearable deformation measurements should not be described as spirometric outputs unless they satisfy the corresponding technical and procedural requirements.

Abnormal respiratory events also require more information than cycle counting. Apnea, hypopnea, and irregular breathing may appear as interrupted waveforms, reduced amplitude, or altered inspiratory–expiratory timing, but different pathological mechanisms can produce similar patterns in a single channel. Distinguishing obstructive from central sleep apnea, for example, requires simultaneous assessment of airflow and respiratory effort. Single-channel flexible devices are therefore more suitable for screening and trend monitoring than for independent clinical classification.

### 5.2. Thoracoabdominal Strain Monitoring

Thoracoabdominal expansion provides a direct mechanical input for flexible strain sensors. Chu et al. placed wearable sensors on the chest and abdomen and compared local deformation with continuous spirometry, demonstrating that strain signals can support respiratory rate and volume estimation under defined conditions [123]. The same study also showed that the relationship between local deformation and ventilation changes with activity, confirming that quantitative volume estimation remains dependent on placement, posture, and individual calibration.

For respiratory applications, the main limitation of piezoresistive strain channels is often low-frequency drift rather than insufficient sensitivity. Viscoelastic relaxation of the substrate, cracks, or conductive networks may occur on a time scale similar to respiration, gradually changing the baseline and waveform amplitude. Stable recovery and low hysteresis are therefore more important than a very high gauge factor measured under rapid stretching.

Capacitive strain channels may provide smoother cyclic responses, but small respiratory signals are easily affected by parasitic capacitance, garment relaxation, and relative displacement between the sensor and the body. Multi-site layouts can reduce dependence on a single sensing location only when the spatial arrangement and fusion method remain stable. Increasing the number of sensors does not compensate for inconsistent placement.

Initial pre-strain also controls how chest-wall motion reaches the sensitive region. Insufficient tension allows fabric or substrate slack to absorb weak deformation, whereas excessive tension may restrict movement and reduce comfort. Postural changes further alter the direction and distribution of thoracoabdominal strain. For prolonged respiratory recordings, attachment tension and body position should be documented throughout the reported on-body session so that changes in respiratory deformation can be distinguished from garment relaxation or general trunk motion. Repeated wear should be reported separately when the sensor is removed and reapplied between recording sessions.

Skin deformation can also be acquired at anatomical locations closer to the airway. Lee et al. developed an ultrathin soft nasal patch that couples respiration-induced nasal deformation to a strain-sensing element and transmits the signal wirelessly during sleep [6]. Comparison with a nasal cannula showed that a conformal local mechanical proxy can reproduce respiratory waveforms without external tubing, while the measurement remains dependent on skin–device mechanics and the relationship between nasal deformation and respiratory airflow.

### 5.3. Pressure, Vibration, and Mechano-Acoustic Monitoring

Respiration generates weak pressure and vibration around the chest wall and upper airway. These signals are smaller and more frequency-dependent than large thoracoabdominal deformation, making their acquisition highly dependent on interface coupling, bandwidth, and anatomical placement. Increasing low-pressure sensitivity may improve signal amplitude, but it can also amplify variations in wearing pressure and body movement.

Piezoelectric and triboelectric devices are well suited to dynamic respiratory vibrations and acoustic events because they respond strongly to changing mechanical inputs. Their output under slow or nearly static chest-wall displacement is less reliable, and humidity or contact-state changes may further affect triboelectric signals. In respiratory monitoring, these devices are therefore more appropriate for event and rhythm detection than for absolute deformation measurement.

Skin-mounted mechano-acoustic systems can extend respiratory monitoring beyond cycle timing by capturing airway and laryngeal vibrations. Kang et al. placed wireless sensors at the sternal notch and recorded respiratory and swallowing biomechanics in healthy participants and patients with dysphagia, with comparison against clinical reference systems [124]. The study demonstrated that a single anatomical location can provide rich cardiopulmonary and laryngeal information, but it also showed that useful classification depends on stable placement, synchronized channels, and separation of overlapping events.

Chest vibration contains both respiratory and cardiac components, which limits simple frequency filtering. When the two rhythms overlap or vary over time, a fixed band-pass range may attenuate the target signal together with the interference. Multi-location sensing or synchronized bioelectrical references may improve separation, but these approaches require stable time alignment and should be justified by the specific measurement problem.

### 5.4. Humidity-Based Respiratory Sensors

Inhalation and exhalation produce periodic humidity changes near the mouth and nose. Humidity sensors can therefore detect airflow alternation without relying on chest-wall deformation and can be integrated into masks or facial patches. Their output reflects local water-vapor transport rather than actual airflow volume, so they are primarily suited to respiratory-cycle detection rather than uncalibrated ventilation measurement.

Hydrophilic sensing layers change resistance or capacitance after water adsorption, while porous architectures shorten diffusion pathways and improve response speed. Chen et al. developed a nanoforest humidity sensor that integrated a sensing capacitor, reference capacitor, thermistor, and microheater [125]. More recent work has extended humidity sensing toward integrated and energy-autonomous respiratory platforms. Zhang et al. combined a flexible MXene-based humidity sensor with micro-supercapacitors in a biodegradable electronic skin and demonstrated real-time monitoring of mouth- and nose-related breathing patterns [126]. This integration addresses power autonomy and material disposal, although the humidity signal remains a local water-vapor proxy rather than a direct measure of ventilation. The reference elements compensated for parasitic and thermal effects, while active heating promoted moisture release, demonstrating that respiratory humidity monitoring benefits from combined material, compensation, and recovery design.

Strong moisture adsorption increases signal amplitude but may also prolong recovery and cause adjacent cycles to overlap. Environmental humidity further determines the available dynamic range: high humidity moves the sensing layer toward saturation, while low temperature may cause condensation and slow desorption. Sensor-airway distance also affects local humidity amplitude, so results obtained at a fixed position cannot be generalized directly to speaking, coughing, mask displacement, or mouth breathing.

Evaluation should therefore include both response and recovery behavior across realistic humidity and temperature conditions. A rapid adsorption response alone is insufficient if the baseline accumulates during repeated breathing. Layer thickness, pore structure, and active recovery should be optimized according to the intended respiratory frequency rather than a single humidity step test.

### 5.5. Temperature-Based Respiratory Sensors

Oronasal temperature sensors estimate respiratory rate from the thermal difference between inhaled and exhaled air. Their performance is governed mainly by thermal time constant, environmental temperature, and sensing position. Low thermal mass improves the ability to resolve rapid cycles but also increases sensitivity to external airflow and device movement, whereas thicker encapsulation improves durability at the cost of slower equilibration.

The usable signal decreases when ambient temperature approaches exhaled-air temperature. Facial motion and bending of the device may further introduce nonthermal changes into the electrical output. Reference elements or differential measurements can reduce baseline drift, but they cannot fully correct changes in airflow caused by sensor displacement.

Combining temperature and humidity channels may improve respiratory-cycle availability because the two pathways fail under different environmental conditions. The purpose of this combination is to reduce dependence on a single proxy signal rather than to increase the number of sensing elements without a defined measurement benefit. A related design direction is to separate multiple respiratory-relevant stimuli at the device level before signal fusion. Song et al. integrated pressure, temperature, and gas-sensing modules using distinct transduction pathways within a flexible respiratory platform [127]. This architecture illustrates how multimodal integration can reduce signal ambiguity when each channel retains a defined physical origin, although the physiological claim supported by each output still depends on appropriate calibration and validation.

### 5.6. Thoracic Bioimpedance and Derived Respiratory Signals

Thoracic bioimpedance measures respiration-related changes in electrical current pathways through the chest. Lung air content and thoracic geometry jointly influence the recorded impedance, allowing respiratory rate to be estimated using electrodes that may also support ECG recording. The signal is nevertheless affected by chest-wall motion, body-fluid distribution, electrode placement, and posture, so it is not a unique measure of lung volume.

Reattachment or postural change alters the current pathway and may invalidate a previously established calibration. Thoracic bioimpedance is therefore more appropriate for respiratory trend monitoring than for uncalibrated absolute tidal volume estimation. Its value in multimodal patches lies partly in providing an independent respiratory channel rather than replacing direct airflow measurement.

Respiration can also be extracted from modulation of ECG waveforms and heart intervals. Roberts et al. developed an open-source respiratory rate algorithm based on single-lead ECG and reported agreement with reference respiration in spontaneously breathing adults [128]. This approach avoids an additional sensing channel, but its performance depends on stable ECG morphology and cardiopulmonary coupling. Motion, arrhythmia, and electrode displacement may reduce or remove the respiratory modulation.

Photoplethysmography can also provide respiration-related information through respiratory modulation of an optically acquired pulse waveform. Its primary signal formation depends on light-tissue interaction and photodetection, which are not reviewed as a main sensing pathway in this article. PPG-derived respiration is therefore included only to illustrate respiratory information derived from an optical cardiovascular signal and is not used as evidence for the performance of the sensing technologies reviewed in detail here.

### 5.7. Performance Evaluation and Key Challenges

Respiratory sensor evaluation should be determined by the intended output. Respiratory rate can be compared with validated airflow or respiratory-effort references, whereas tidal volume requires calibrated flow or volume measurement. Systems intended to detect sleep-related respiratory events should be validated against polysomnography because airflow, respiratory effort, oxygenation, and sleep state are required for clinical classification. The 2026 nasal-patch study further illustrates this distinction because close reproduction of respiratory waveforms relative to a cannula supports the recorded respiratory pattern, while stronger claims concerning ventilation or clinical sleep-event classification would require references matched to those outputs [6].

High correlation with a reference does not establish agreement for an absolute respiratory output. Respiratory rate or tidal volume estimates should therefore report measurement error and agreement, including systematic bias and limits of agreement when appropriate. Event-detection claims should use predefined event definitions and report sensitivity together with false-positive or positive-predictive performance. Long-duration recordings should separately report the proportion of time for which a valid respiratory output is available. Bujan et al. assessed both instantaneous and overnight respiratory rate results in a continuous monitoring system, providing a useful methodological example for agreement reporting, although the contactless device itself is not evidence for the performance of flexible sensors [129].

Motion artifacts differ across sensing pathways. Thoracoabdominal strain channels are affected by trunk motion, oronasal sensors are sensitive to position and local airflow, and bioimpedance depends on stable electrode contact. Sensor displacement, detachment, and saturation should be identified and reported as invalid respiratory intervals rather than processed as ordinary waveform noise.

Validation should include natural activity and irregular respiration rather than only instructed breathing in seated participants. Walking, postural change, speaking, coughing, and breath holding may all disrupt periodicity or generate similar signals. Studies should report invalid periods and failed measurements instead of presenting only selected segments with successful output.

Respiratory studies intended to support long-term use should report the actual on-body duration and acquisition pattern, together with periods in which valid respiratory output is unavailable. Nominal wear time should be distinguished from effective valid data time when signal loss, device removal, or acquisition interruption occurs.

The respiratory sensing approaches reviewed here differ mainly in how directly their measured signal represents the physiological quantity being reported. A signal that is sufficient for identifying respiratory timing may not retain the information needed for estimating volume or classifying a clinical respiratory event. Increasing sensor sensitivity cannot recover information that is absent from the selected proxy or lost because of poor coupling and motion. Validation should therefore be matched to the intended respiratory output, and complementary measurements become necessary when that output depends on physiological information that a single sensing pathway cannot provide. Table 2 compares representative studies by separating reported device performance from on-body monitoring evidence and reference-based validation. Numerical values are retained when the original studies report directly comparable quantities, including sensitivity, detection limit, response time, monitoring duration, measurement bias, or agreement statistics, while unreported quantities are not inferred from qualitative demonstrations.

The numerical values in Table 2 should be interpreted within the conditions under which they were obtained. High sensitivity, low detection limits, or extensive mechanical cycling characterize device performance but do not establish physiological accuracy when reference comparison is absent. Conversely, quantitative agreement with a physiological reference addresses a stronger measurement claim even when the underlying sensor does not exhibit the largest nominal device-level response. The comparison therefore separates technical performance from evidence supporting the reported physiological output rather than ranking devices by a single metric.

## 6. Conclusions and Outlook

Across the sensing modalities examined in this review, the recorded output is shaped by more than the intrinsic response of the sensing material. Physiological information may be altered before transduction by tissue and interface conditions, and further distortion can arise from environmental exposure, motion, device drift, or loss of coupling during wear. These effects become increasingly important when a local sensor output is used to infer a physiological quantity that the device does not measure directly. The common challenge is therefore to preserve and demonstrate a reproducible relationship between the physiological process of interest and the reported output under the conditions in which the system is intended to operate.

### 6.1. From Device Response to Measurement Validity

**Figure 5:**
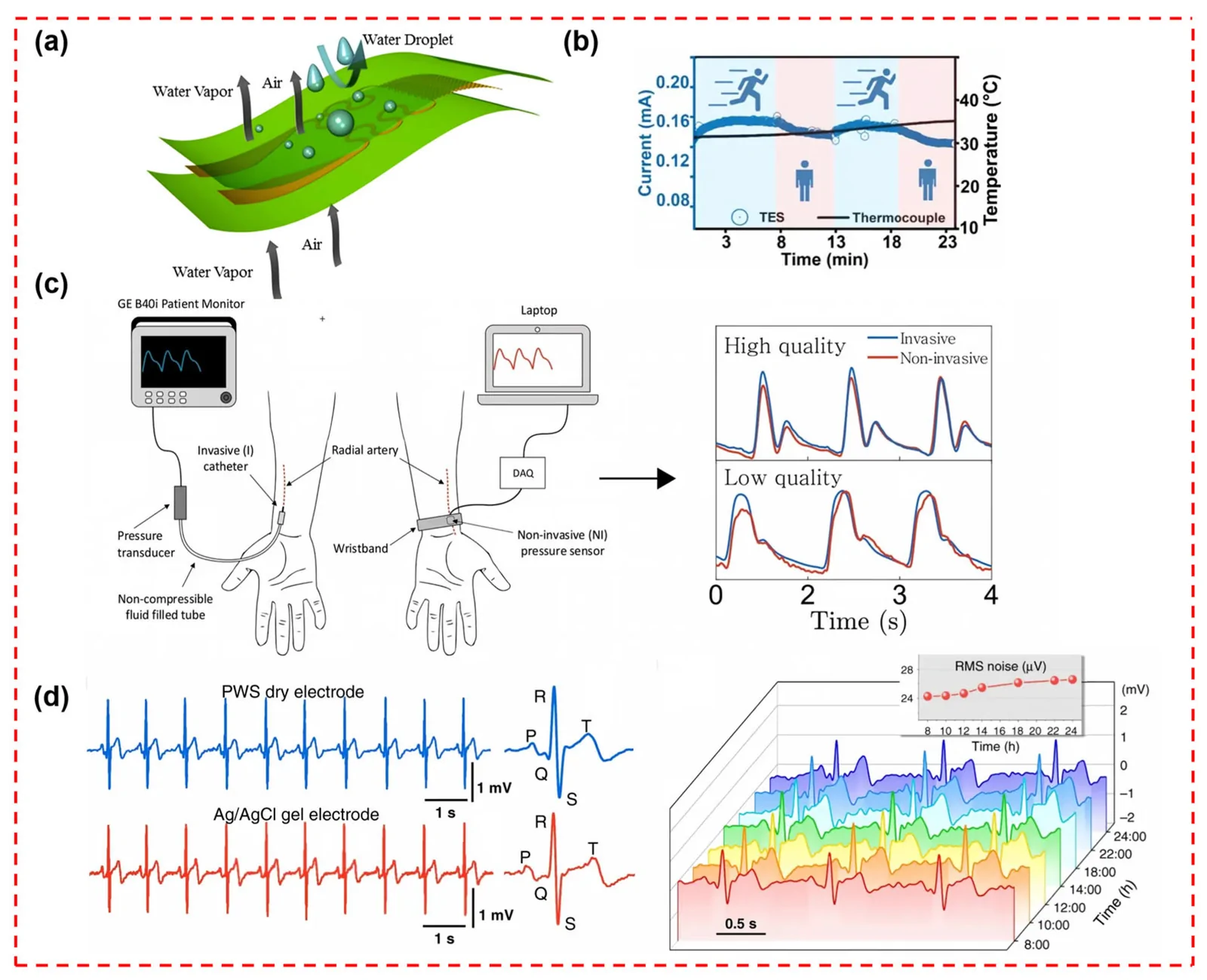
From interface engineering to measurement validity in skin-interfaced flexible sensors. (**a**) Breathable semipermeable encapsulation enabling water-vapor transport while maintaining skin-compatible attachment for continuous temperature monitoring. Adapted from Ref. [64]. (**b**) Strain-insensitive thermistor electronics enabling reliable temperature monitoring during body motion. Adapted from Ref. [53]. (**c**) Comparison between wearable pulse sensing and invasive arterial reference measurement, illustrating the importance of reference-based validation for physiological waveform interpretation. Adapted from Ref. [102]. (**d**) ECG assessment using self-adhesive dry electrodes after prolonged on-skin wear, including 16 h of continuous wear and additional signal quality evaluation at longer follow-up intervals. Adapted from Ref. [119].

The measurement process described in this review also clarifies where different forms of validation become necessary. Verification concerns the performance of the sensor product and its ability to produce the intended technical output, whereas analytical validation addresses the accuracy of the physiological measure derived from that output. Clinical validation considers whether the measure is associated with the clinical concept of interest within the specified context of use. These evidence levels address different questions from the physical measurement sequence, but together they prevent device response, physiological measurement, and clinical interpretation from being treated as equivalent claims. At the device and interface level, breathable encapsulation can preserve water-vapor transport during temperature monitoring (Figure 5a), while strain-insensitive thermistor design can reduce mechanically induced error before signal interpretation (Figure 5b). These engineering improvements do not replace physiological validation, as illustrated by comparison of a wearable pulse waveform with an invasive arterial reference (Figure 5c) and by prolonged ECG assessment showing that interface stability must also be maintained over the intended wearing period (Figure 5d).

Evidence should therefore remain proportional to the claim being made. Successful signal acquisition can demonstrate technical feasibility, but a derived physiological measure requires comparison with an appropriate reference, and a clinical claim requires evidence obtained in the relevant population and context of use. Reporting should make these distinctions explicit rather than using high sensitivity, correlation, or a limited human demonstration as interchangeable evidence of measurement validity.

The statistical evidence should also be matched to the type of physiological output being evaluated. For absolute quantitative outputs, agreement with a reference should be assessed from paired measurement error rather than correlation alone. Event-detection and classification claims require accuracy measures based on correctly and incorrectly identified events or cases, while trend and waveform outputs require evidence that physiologically relevant changes, timing, or morphology are preserved. Because these outputs pose different validation questions, no single accuracy statistic is sufficient across all sensing modalities [130,131,132]. Quantitative comparison is therefore performed using output-specific metrics rather than by forcing heterogeneous sensing modalities into a single numerical ranking. Table 3 summarizes the minimum validation evidence recommended for these output types.

### 6.2. Dynamic Skin–Device Interfaces

Across the reviewed sensing pathways, changes in skin–device coupling can alter the relationship between the physiological source and the recorded signal even when the sensing material remains functional. Interface variation should therefore be treated as a time-dependent part of the measurement chain rather than only as an attachment problem. Future systems should either stabilize this transfer relationship or use an appropriate interface or signal quality indicator to identify conditions in which the established calibration may no longer apply. Reference channels and interface self-checks are most useful when they support predefined decisions about signal validity or recalibration rather than serving only as retrospective indicators of artifact [131,133,134].

### 6.3. Long-Term Reliability

Because skin–device coupling evolves during wear, reliability claims should be tied to the temporal evidence actually reported. Human studies should distinguish nominal wear time, active acquisition time, and effective valid data time while documenting interruptions, reattachment, recalibration, and skin reactions that occur during the monitoring period. Repeated-session follow-up should be identified separately from uninterrupted acquisition, and material cycling or environmental aging should remain classified as durability evidence rather than on-body monitoring evidence. Table 4 summarizes the corresponding reporting requirements.

### 6.4. Multimodal Signal Decoupling

Multimodal sensing is useful when different channels resolve a defined measurement ambiguity. The value of an additional channel is reduced when the same body motion, attachment change, or environmental disturbance contaminates multiple measurements through a shared error pathway. Sensor selection should therefore prioritize complementary physiological information and error pathways rather than maximum channel count. Decoupling should begin by identifying which components of each channel are linked to the target physiology and which are associated with shared disturbance. A multimodal system should then demonstrate that the added channel contributes information unavailable from the primary measurement or provides an independent indicator of disturbance that improves interpretation [4,5,127,157]. Recent cross-modal epidermal, self-decoupled ionic, and respiratory sensing systems illustrate different hardware-level routes to this goal, ranging from fusion of electrical and mechanical information to intrinsic separation of mechanically, thermally, and chemically driven responses. These architectures reduce specific forms of measurement ambiguity, but their value should ultimately be assessed by whether decoupling improves the validity and robustness of the intended physiological output.

Hardware-level strategies should reduce measurement ambiguity before algorithmic fusion is relied upon to separate residual interference. Algorithms may suppress remaining artifacts, but they cannot reconstruct physiological information lost through detachment, saturation, or severe waveform distortion. Fusion should therefore account for channel quality and availability rather than assume that every input remains equally informative throughout wear. Validation should compare the multimodal output with an appropriate single-channel or reduced-channel baseline under matched conditions and examine performance when an input becomes degraded or unavailable. Machine-learning models should also be tested across participants, devices, and usage conditions so that apparent performance gains are not driven primarily by placement or batch differences [157].

These considerations also extend from individual sensing channels to the complete wearable system. System-level validation should evaluate concurrent sensing, processing, storage, and communication because interactions among these functions can alter sampling continuity, synchronization, local heat generation, or wearability. Reliability should therefore be judged from the synchronized physiological information delivered over the intended use period rather than from isolated sensor performance measured with external laboratory instrumentation.

### 6.5. Clinical Translation

Clinical claims should match the level of evidence. Trend monitoring requires stable detection of meaningful change, screening requires evaluation of false-negative and false-positive results, and diagnostic support requires validation in the target patient population. The V3+ framework further emphasizes usability validation, recognizing that accurate technology has limited value if intended users cannot deploy it correctly and consistently [158,159].

Future studies should define the intended physiological quantity and context of use before optimizing device performance because these choices determine the required interface stability, reference method, monitoring conditions, and acceptable measurement failure. Evaluations conducted under realistic activity and wearing conditions can then establish whether the relationship observed in controlled experiments persists in the population for which the device is intended. For multimodal systems, clinical validation should establish whether the additional channels improve the intended physiological or clinical output over an appropriate simpler configuration rather than assume that more simultaneously recorded signals provide greater clinical value.

Progress in skin-interfaced flexible sensing should therefore be judged by whether the complete measurement process preserves physiologically meaningful information with sufficient reliability for the intended claim. Separating the directly measured quantity from the derived physiological metric and from any subsequent clinical interpretation makes the available evidence easier to assess and reduces overinterpretation of strong device-level performance. This perspective allows advances in sensing materials and device engineering to be considered together with the evidence required for reliable physiological measurement and eventual clinical use.

## Figures and Tables

**Table 1:** Direct measurands, supported physiological outputs, inferential boundaries, and reference requirements across skin-interfaced sensing modalities.

Signal Domain	Direct Measurand at the Skin	Potentially Supported Output	Claim Requiring Further Validation	Appropriate Reference or Validation Requirement	Ref.
Skin temperature	Local surface temperature	Site-specific thermal trend; spatial temperature difference	Core temperature; quantitative perfusion; disease identification	Calibrated contact thermometer or thermal imaging for local temperature; appropriate core-temperature reference for core estimates	[18,19,20]
Arterial pulse	Local pressure or displacement waveform	Heart rate; beat timing; relative waveform morphology	Absolute blood pressure; pulse-wave velocity; cardiac output or other hemodynamic variables	Simultaneous ECG for beat timing; tonometry or invasive pressure waveform for morphology; clinical investigation consistent with ISO 81060-3 [21] for continuous blood pressure claims	[22,23]
Joint motion	Local skin strain, bending, or curvature	Movement cycles; relative range-of-motion trend	Absolute joint angle; clinical mobility score	Goniometer or optical motion-capture system under matched movement conditions	[24,25,26]
Muscle mechanical activity	Local deformation, pressure, or vibration	Contraction timing; relative mechanical change	Muscle force; fatigue severity; neuromuscular diagnosis	Dynamometry, ultrasound, or reference EMG according to the intended output	[27,28]
ECG	Skin potential difference produced by cardiac electrical activation	Rhythm; event timing; waveform morphology	Diagnostic classification or clinical decision	Simultaneous clinical ECG with matched lead placement, bandwidth, sampling rate, and filtering	[29,30,31]
sEMG	Surface potential representing summed motor-unit action potentials	Activation timing; relative amplitude; spectral change	Muscle force; quantitative fatigue; neuromuscular diagnosis	Conventional Ag/AgCl sEMG with matched placement and spacing; dynamometry for force-related claims	[32,33,34]
EOG	Periocular potential change associated with rotation of the corneo-retinal dipole	Eye-movement timing; approximate direction; blink detection	Precise gaze position; saccade amplitude; sleep-stage or neurological classification	Synchronized video eye tracking or laboratory EOG with matched electrode placement	[35,36,37]
EDA	Time-dependent skin conductance under a defined excitation mode	Skin conductance level and skin conductance responses within a defined context	Identification of a specific emotion, stress disorder, or disease	Reference EDA system with matched site and excitation mode; standardized EDA acquisition and reporting procedures	[38,39,40]

**Table 2:** Quantitative performance, monitoring evidence, reference validation, and remaining evidence gaps in representative flexible sensing platforms.

Modality	Representative Platform	Key Reported Performance	On-Body Duration and Acquisition Pattern	Reference and Validation Evidence	Main Evidence Boundary	Ref.
Temperature	Nacre-inspired MXene laminated thermistor	−1.32% °C^−1^; ≈0.3 °C resolution; 20,000 folding cycles	Sustained on-body duration not reported; 20,000 cycles were mechanical folding tests rather than physiological monitoring	Dynamic exercise comparison with a commercial thermocouple	Measures local temperature; mechanical folding durability does not establish performance during extended on-body monitoring	[53]
Pressure	Hollow-sphere polypyrrole piezoresistive film	Detection limit <1 Pa; rapid and repeatable response	No sustained on-body monitoring duration reported; laboratory and prototype demonstrations only	Laboratory and prototype demonstrations	Low-pressure detectability does not establish pulse-wave or hemodynamic accuracy	[82]
Pressure	Graded intrafillable iontronic sensor	3302.9 kPa^−1^ below 10 kPa; 0.08 Pa limit; 9 ms response; 5000 cycles	No sustained on-body duration reported; 5000-cycle testing reflects device durability	Physiological-signal demonstrations	Strong frequency, ionic, temperature, and encapsulation dependence; no clinical metric validation	[92]
Biopotential	PEDOT:PSS/WPU/sorbitol self-adhesive dry electrode	ECG, EMG, and EEG under dry, wet, and moving conditions; 16 h skin wear	16 h continuous wear; additional ECG quality assessments were reported at later follow-up intervals	Ag/AgCl comparison; atrial-fibrillation and muscle-function demonstrations	Clinical evidence was limited; broader population and repeated-wear testing required	[119]
Biopotential	Stretchable microneedle adhesive patch	Reduced contact impedance without skin preparation, including contaminated-skin conditions	Explicit sustained on-body recording duration not reported in the reviewed study	ECG/EMG acquisition during movement and exoskeleton control	Slightly invasive interface; comfort, barrier recovery, and repeated-use safety require assessment	[120]
EDA	Submicrometre graphene electronic tattoo with heterogeneous serpentine interconnect	50-fold interconnect strain reduction; approximately 300 nm tattoo thickness	Three 15 h nonstop ambulatory EDA monitoring sessions	Ambulatory palmar EDA with comparison to reference measurements	EDA reflects autonomic arousal but is not specific to an emotion or disease	[28]
Respiration	Ribcage-abdomen piezoresistive strain sensors	Respiratory rate and volume estimation from ribcage and abdominal strain signals	2 h wireless wear session with 2 min respiratory recordings performed approximately every 15 min	Spirometer comparison; volume bias 0.0347 L; limits of agreement −0.276 to 0.346 L; CCC 0.956 for exhalation	Volume estimation required participant-specific calibration and remained activity-dependent	[123]
Respiration and swallowing	Soft suprasternal mechano-acoustic sensor	Continuous wireless vibration recording using differential operation	Sustained recording duration not explicitly reported; the adhesive was designed to support wear for up to 12 h	Healthy adults, *n* = 67; patients with dysphagia, *n* = 4; clinical-reference comparison	Small patient cohort; performance depends on location and event-separation algorithms	[124]
Respiratory humidity	Nanoforest capacitive sensor with reference capacitor, thermistor, and microheater	Eightfold sensitivity improvement; recovery reduced from 11.1 to 5 s by active heating	Short mask-based respiration demonstrations; sustained on-body duration not reported	Mask-based respiration demonstrations and repeated measurements	Humidity is not airflow volume; active heating introduces power and thermal-management costs	[125]
Arterial pulse/blood pressure	Battery-free wireless epidermal sensor network	Continuous systolic blood pressure monitoring with battery-free epidermal sensing and wireless power/data transfer	40 min continuous commute monitoring; 6.5 h continuous sleep monitoring; additional continuous monitoring during strenuous exercise	Dynamic physiological testing including exercise; reference-based systolic blood pressure evaluation	System integration supports continuous monitoring, but blood pressure validity depends on the reference protocol and tested physiological range	[3]
Respiration	Soft skin-interfaced nasal strain patch	Wireless acquisition of respiration-induced nasal deformation during sleep	Continuous respiratory recording during sleep; exact on-body monitoring duration not reported	Comparison with gold-standard nasal cannula respiratory measurements	Measures nasal deformation as a respiratory proxy; ventilation and clinical event classification require output-specific validation	[6]

**Table 3:** Recommended validation evidence for different physiological output types.

Output Type	Recommended Validation Evidence	Reporting Focus	Ref.
Absolute continuous quantity	Bias; limits of agreement; uncertainty estimates	Agreement with reference; predefined acceptance limits	[131]
Event detection	Sensitivity; PPV or false-alarm rate; detection latency	Predefined event and matching rules	[133]
Classification output	Sensitivity; specificity; PPV/NPV; class-specific accuracy	Reference standard and target population	[134]
Trend or change monitoring	Within-subject change agreement; concordance; repeatability	Accuracy of physiological change	[135]
Waveform or timing output	Timing error; feature-specific error; repeatability	Synchronized reference and predefined features	[30]

**Table 4:** Minimum reliability tests and reporting items for prolonged and long-term skin-interfaced sensing.

Evaluation Domain	Application-Relevant Test Conditions	Core Metrics to Report	Examples of Predefined Invalid or Adverse Triggers	Recommended Design or Reporting Response	Ref.
Skin–device interface	Sweating, friction, multidirectional motion, repeated attachment, and intended wear duration	Adhesion loss; interface impedance or contact-pressure drift; partial or complete detachment events	Partial or complete detachment; abrupt contact change; interface value outside validated range	Use conformal mechanics, moderate adhesion, and low-traction interconnections; report signal-loss and reattachment periods	[136,137,138]
Transducer and material stability	Application-matched temperature, humidity, strain, loading rate, frequency, and repeated-use conditions	Sensitivity drift; hysteresis; baseline recovery; bandwidth; device-to-device and batch variability	Saturation; irreversible baseline shift; calibration error beyond predefined tolerance	Test combined rather than isolated stressors; report device number, fabrication batches, and uncertainty intervals	[139,140,141]
Signal and algorithm validity	Intended posture, activity, placement, environment, monitoring duration, and target population	Output-specific validity metrics; between-session agreement; valid data fraction	Signal quality index below predefined threshold; output outside validated operating domain	Use synchronized reference measurements, external validation, and explicit signal quality indicators	[142,143,144]
Reattachment and recalibration	Removal, repositioning, anatomical-site variation, and repeated wearing sessions	Baseline shift; recalibration frequency; recovery time; between-session agreement	New baseline cannot be linked to previous calibration; error exceeds intended-use limit	Provide placement guidance and interface self-checks; report whether recalibration was required	[145,146,147]
Integrated-system reliability	Complete operation with battery, processor, wireless unit, connectors, packaging, and algorithms	Local heating; power consumption; packet loss; synchronization error; system uptime	Data interruption; channel desynchronization; thermal or power limitation exceeded	Validate complete wearable systems rather than isolated sensors; maintain synchronized timestamps and failure logs	[148,149,150]
Skin tolerance and usability	Intended wear duration, repeated removal, sweating, daily activity, and target-user deployment	Erythema; discomfort; maceration; removal injury; setup errors; adherence; premature removal	Clinically relevant skin reaction; intolerable discomfort; repeated placement or setup failure	Use breathable interfaces and atraumatic removal; perform usability and target-population validation	[151,152,153]
Monitoring duration and continuity	Intended wear duration and repeated-use conditions	On-body duration; acquisition pattern; valid data time	Signal loss; device removal; acquisition interruption	Separate wear time, acquisition time, and valid data time; distinguish repeated sessions from continuous recording	[154,155,156]

## Data Availability

Not applicable.
